# Quercetin Enhances Topotecan Cytotoxicity in Retinoblastoma Cells Through ROS-Associated Stress and Apoptotic Signaling

**DOI:** 10.3390/biom16040597

**Published:** 2026-04-17

**Authors:** Aydın Maçin, Erkan Duman, İlhan Özdemir, Mehmet Cudi Tuncer

**Affiliations:** 1 Department of Ophthalmology, Diyarbakır Private Batı Hospital, Diyarbakır 21100, Turkey; 2 Department of Ophthalmology, WestEye Private Hospital, Erbil 44001, Iraq; ophthalmo48@outlook.com; 3 Department of Histology and Embryology, Faculty of Medicine, Kahramanmaraş Sütçü İmam University, Kahramanmaraş 46040, Turkey; ilhanozdemir25@yandex.com; 4 Department of Anatomy, Faculty of Medicine, Dicle University, Diyarbakır 21280, Turkey; drcudi@hotmail.com

**Keywords:** quercetin, topotecan, retinoblastoma, signaling pathway, synergy, apoptosis

## Abstract

Quercetin, a naturally occurring flavonoid, exhibits antiproliferative and pro-apoptotic effects across various cancer models. Topotecan, a topoisomerase I inhibitor, is used in the treatment of retinoblastoma; however, its clinical utility is limited by dose-dependent toxicity. This study aimed to investigate whether quercetin is associated with enhanced topotecan-induced cytotoxicity in retinoblastoma and to explore the underlying mechanisms under both two-dimensional (2D) and three-dimensional (3D) conditions. Cell viability was assessed using the MTT assay, and drug interactions were evaluated using the combination index (CI) based on the Chou–Talalay method. Apoptosis was analyzed by Annexin V-FITC/PI staining and flow cytometry. Reactive oxygen species (ROS) levels and mitochondrial membrane potential were evaluated using fluorometric methods, and N-acetyl-L-cysteine (NAC) was used for functional modulation of oxidative stress. Three-dimensional tumor spheroid models were used to assess treatment effects under conditions that partially recapitulate tumor architecture. Gene expression levels of apoptosis-related markers and PI3K/Akt/mTOR pathway components were analyzed by quantitative real-time polymerase chain reaction (qRT-PCR). The combination of quercetin and topotecan was associated with synergistic cytotoxic effects in Y79 cells (CI < 1), accompanied by increased ROS levels, mitochondrial membrane depolarization, and elevated apoptotic cell death. NAC co-treatment partially attenuated ROS levels and restored cell viability. In 3D spheroid models, combination treatment induced structural disruption, reduced viability, and increased cell death, effects that were partially reversed by NAC. Gene expression analysis revealed upregulation of pro-apoptotic genes and downregulation of survival-related genes, along with increased PTEN expression. Quercetin is associated with enhanced topotecan-induced cytotoxicity in retinoblastoma cells under both 2D and 3D conditions. These effects were associated with ROS-associated cellular stress, mitochondrial dysfunction, and modulation of apoptotic and survival-related pathways. The partial rescue by NAC supports a contributory, but not exclusive, role of oxidative stress. These findings should be interpreted within a preclinical context and suggest that quercetin may represent a potential adjunct strategy warranting further validation in translational and in vivo models.

## 1. Introduction

Among pediatric ocular malignancies, retinoblastoma holds the distinction of being the most prevalent intraocular tumor of childhood, accounting for roughly 3% of all cancers diagnosed in children. It predominantly manifests before the age of five and carries a life-threatening prognosis when diagnosis is delayed [1]. At the molecular level, biallelic loss-of-function mutations in the RB1 tumor suppressor gene are considered the initiating genetic lesion; RB1 maps to chromosomal locus 13q14 and encodes the pRb protein, a master regulator of cell cycle progression [2]. Disruption of pRb function releases cells from normal proliferative constraints, thereby fueling tumor growth. Current management encompasses a range of modalities, including systemic chemotherapy, focal laser photocoagulation, cryotherapy, external beam radiotherapy, and surgical enucleation, selected according to tumor stage and laterality [3].

Of the available chemotherapeutic agents, topotecan has attracted particular attention in the management of retinoblastoma, particularly in the context of localized delivery strategies such as intra-arterial chemotherapy. As a camptothecin-derived topoisomerase I inhibitor, topotecan stabilizes the topoisomerase I–DNA cleavage complex during replication, converting transient single-strand breaks into lethal double-strand DNA damage that ultimately triggers apoptosis [4]. Topotecan has demonstrated antitumor activity against retinoblastoma in both preclinical and clinical settings, either as a single agent or in combination with other chemotherapeutics [3]. In contemporary clinical practice, chemotherapy delivery in retinoblastoma is highly route-dependent and includes intravenous, intra-arterial, periocular, and intravitreal approaches, selected according to tumor burden, laterality, and metastatic risk [5,6]. Among these modalities, intravitreal chemotherapy has emerged as a key strategy for the management of recurrent or refractory vitreous seeds, which remain a major cause of treatment failure following conventional therapies [7]. Recent clinical evidence indicates that intravitreal topotecan is an effective and well-tolerated option in this setting, achieving high rates of vitreous seed regression and globe salvage in long-term follow-up studies [8]. Importantly, localized delivery approaches such as intra-arterial and intravitreal administration enable higher intraocular drug concentrations while minimizing systemic exposure and associated toxicity, whereas systemic administration remains limited due to dose-dependent adverse effects and pharmacokinetic constraints [3,6]. Despite these advances, chemotherapy-related toxicity and tumor recurrence driven by resistant clones remain significant challenges, underscoring the need for optimized combination strategies and novel adjunctive approaches [6]. Accordingly, the precise clinical role, optimal dosing strategies, and combinatorial use of topotecan in retinoblastoma continue to be refined.

Multiple preclinical and clinical investigations have validated its retinoblastoma activity, and intra-arterial administration achieves high intraocular drug exposure while limiting systemic toxicity [9]. Despite this efficacy, dose-dependent adverse effects, including bone marrow suppression, gastrointestinal injury, and genotoxic damage to healthy tissues through ROS overgeneration, constrain its therapeutic window [10]. These limitations motivate the search for combination regimens capable of boosting tumor-cell kill while sparing normal tissues [11]. Quercetin (3,3′,4′,5,7-pentahydroxyflavone), a polyphenolic flavonoid abundant in dietary sources such as onions, apples, grapes, green tea, and broccoli, possesses well-documented antioxidant, anti-inflammatory, antiproliferative, and pro-apoptotic activities across a wide spectrum of cancer types [12]. Its antitumor actions involve simultaneous modulation of several oncogenic cascades, including PI3K/Akt/mTOR, MAPK/ERK, NF-κB, and Wnt/β-catenin [13], and quercetin has also been shown to potentiate the efficacy of established chemotherapeutics while partially reversing drug resistance [14].

The capacity of quercetin to augment topotecan-induced cytotoxicity has been documented in several tumor models. A study in breast cancer lines demonstrated that topotecan exerts its cytotoxic action largely through ROS accumulation and that quercetin, rather than neutralizing this oxidative burden, paradoxically amplified topotecan-mediated cytotoxicity in both cell lines [15]. This seemingly counterintuitive observation raises the possibility that, in the context of combination therapy, quercetin can switch from an antioxidant to a pro-oxidant role, intensifying oxidative pressure in malignant cells [16]. Equally important are reports of tissue-protective actions: a murine bone marrow study found that quercetin attenuated topotecan-induced DNA strand breaks and oxidative injury in a concentration-dependent fashion [17], suggesting a potential for selective augmentation of chemotherapy efficacy in tumor tissue while sparing normal cells. A comprehensive mechanistic review further catalogued quercetin’s adjuvant potential, highlighting apoptosis induction, cell cycle arrest, anti-angiogenic activity, and chemosensitization as contributing mechanisms [18]. Within the specific context of retinoblastoma, however, data remain sparse. The sole published investigation in Y79 cells reported ROS-driven mitochondrial pathway apoptosis following quercetin treatment [19], yet the combined effects of quercetin and topotecan and their impact on non-malignant cell viability via the HaCaT keratinocyte model have not been examined [6]. The present study was therefore designed to address these gaps.

Based on these considerations, this study was designed to investigate whether quercetin is associated with enhanced topotecan-induced cytotoxicity in retinoblastoma within a preclinical experimental framework. Given the clinical limitations of topotecan, particularly dose-dependent toxicity, identifying adjunct strategies that may improve therapeutic efficacy while potentially reducing required drug exposure remains of considerable interest. We hypothesized that quercetin may modulate topotecan-induced cytotoxic responses through a multifactorial mechanism involving ROS-associated cellular stress, mitochondrial dysfunction, and alterations in apoptotic and survival-related signaling pathways. To address this, we evaluated drug interaction dynamics, apoptosis induction, intracellular ROS generation, and mitochondrial membrane potential in Y79 retinoblastoma cells. To improve translational relevance, these effects were further examined in three-dimensional (3D) tumor spheroid models, which partially recapitulate tumor architecture and microenvironmental constraints. In addition, NAC pretreatment experiments were performed to functionally assess the contribution of oxidative stress. Furthermore, the selectivity of the treatment response was evaluated using non-malignant HaCaT cells, and key molecular pathways were analyzed at the transcriptional level. Collectively, this study aims to provide a mechanistically integrated and preclinical evaluation of quercetin as a potential adjunct to topotecan in retinoblastoma, with an emphasis on translational relevance.

## 2. Materials and Methods

### 2.1. Cell Culture

Two cell lines were employed in the experiments: the human retinoblastoma cell line Y79 (ATCC^®^ HTB-18™, Manassas, VA, USA) and the non-cancerous human keratinocyte cell line HaCaT (CLS Cell Lines Service, Eppelheim, Germany). Y79 cells were maintained in RPMI-1640 medium (Gibco, Thermo Fisher Scientific, Waltham, MA, USA) supplemented with 20% fetal bovine serum (FBS, Gibco, Thermo Fisher Scientific, Waltham, MA, USA)), 1% L-glutamine, and 1% penicillin–streptomycin. HaCaT cells were cultured in DMEM (Gibco, Thermo Fisher Scientific, Waltham, MA, USA) supplemented with 10% FBS, 1% L-glutamine, and 1% penicillin–streptomycin.

All cells were maintained at 37 °C in a humidified atmosphere containing 5% CO_2_. Y79 cells were cultured as suspension cells and maintained by regular medium replenishment, whereas HaCaT cells were subcultured upon reaching approximately 80–90% confluency.

### 2.2. Reagents and Antibodies

Quercetin (purity ≥95%, Sigma-Aldrich, St. Louis, MO, USA; Q4951) and topotecan hydrochloride (purity ≥98%, Sigma-Aldrich; T2705) were dissolved in dimethyl sulfoxide (DMSO, Gibco, Thermo Fisher Scientific, Waltham, MA, USA) to prepare stock solutions and subsequently diluted to the desired working concentrations in appropriate culture medium immediately before each experiment. The final DMSO concentration did not exceed 0.1% (*v*/*v*) in any treatment condition. NAC (NAC; Sigma-Aldrich, St. Louis, MO, USA; A9165) was used as a ROS scavenger in antioxidant rescue experiments. For immunocytochemical analysis, a rabbit polyclonal anti-caspase-9 antibody (Abcam, Cambridge, UK; ab202068) was used as the primary antibody, followed by an HRP-conjugated secondary antibody.

### 2.3. Cell Viability and Cytotoxicity

Cell viability was assessed using the MTT [3-(4,5-dimethylthiazol-2-yl)-2,5-diphenyltetrazolium bromide] colorimetric assay. Y79 and HaCaT cells were seeded in 96-well plates at a density of 1 × 10^4^ cells per well. Due to their suspension nature, Y79 cells were allowed to stabilize after seeding, whereas HaCaT cells were allowed to adhere overnight. Cells were treated with increasing concentrations of quercetin (0, 10, 25, 50, 100, and 200 µM) and topotecan (0, 5, 10, 25, 50, and 100 µM) for 24 h and 48 h.

Following treatment, 10 µL of MTT solution (5 mg/mL) was added to each well and incubated for 4 h at 37 °C. The resulting formazan crystals were dissolved in 100 µL DMSO, and absorbance was measured at 570 nm using a microplate reader. Cell viability was expressed as a percentage relative to untreated control cells. All experiments were performed in triplicate and repeated in at least three independent experiments. MTT assays were performed under standardized conditions with consistent reagent preparation and incubation parameters across all groups.

### 2.4. Combination Index and Synergy Analysis

Drug interactions between quercetin and topotecan were evaluated using the CI method based on the Chou–Talalay algorithm. Cells were treated with fixed-ratio combinations of quercetin and topotecan (1:1, 1:2, and 2:1), and viability data obtained from MTT assays were used for analysis.

CI values were calculated using CompuSyn software (CompuSyn, Inc., Paramus, NJ, USA; version 1.0). CI < 1 indicates synergism, CI = 1 indicates an additive effect, and CI > 1 indicates antagonism. These analyses enabled a quantitative assessment of the degree of interaction between the two agents across different effect levels. CI analysis was performed across concentration ranges derived from dose–response experiments (quercetin: 10–200 µM; topotecan: 5–100 µM) using fixed ratios (1:1, 1:2, and 2:1).

### 2.5. Apoptosis Analysis (Annexin V-FITC/PI Staining)

Apoptotic cell death was quantified using dual-parameter flow cytometry with an Annexin V-FITC/PI apoptosis detection kit (BD Biosciences, San Jose, CA, USA). Y79 cells were seeded in 6-well plates at a density of 5 × 10^5^ cells per well and treated with quercetin (36.7 µM), topotecan (5.8 µM), or their combination for 24 h and 48 h.

Following treatment, cells were collected, washed twice with cold phosphate-buffered saline (PBS), and resuspended in 100 µL of binding buffer. Annexin V-FITC (5 µL) and propidium iodide (PI, 5 µL) were added to each sample, followed by incubation for 15 min at room temperature in the dark.

Samples were analyzed using a FACSCalibur flow cytometer (BD Biosciences, San Jose, CA, USA), and data were processed with CellQuest Pro software (BD Biosciences, San Jose, CA, USA; version 5.2.1). Cell populations were classified as viable (Annexin V^−^/PI^−^), early apoptotic (Annexin V^+^/PI^−^), late apoptotic (Annexin V^+^/PI^+^), and necrotic (Annexin V^−^/PI^+^).

### 2.6. Measurement of Reactive Oxygen Species

Intracellular ROS levels were assessed using the cell-permeant fluorescent probe 2′,7′-dichlorodihydrofluorescein diacetate (DCFH-DA; Sigma-Aldrich, St. Louis, MO, USA; D6883). Y79 cells were seeded in 96-well plates at a density of 2 × 10^4^ cells per well and allowed to stabilize for 24 h prior to treatment.

Cells were then treated with quercetin (36.7 µM), topotecan (5.8 µM), or their combination for 48 h. To evaluate the contribution of oxidative stress, NAC (NAC; 5 mM) was used as an ROS scavenger in parallel experimental groups, including combination + NAC conditions.

Following treatment, cells were washed with PBS and incubated with 10 µM DCFH-DA in serum-free medium for 30 min at 37 °C in the dark. Fluorescence intensity was measured at excitation/emission wavelengths of 485/528 nm using a microplate reader. Fluorescence measurements were performed under identical conditions across all experimental groups to ensure comparability.

ROS levels were expressed as percentages relative to untreated control cells. All experiments were performed in triplicate and repeated in at least three independent experiments.

### 2.7. Mitochondrial Membrane Potential (ΔΨm) Measurement

Mitochondrial membrane potential (ΔΨm) was evaluated using the voltage-sensitive cationic dye JC-1 (5,5′,6,6′-tetrachloro-1,1′,3,3′-tetraethylbenzimidazolylcarbocyanin iodide; Sigma-Aldrich, St. Louis, MO, USA; T4069). Y79 cells were seeded in 96-well plates at a density of 2 × 10^4^ cells per well and allowed to stabilize for 24 h prior to treatment.

Cells were treated with quercetin (36.7 µM), topotecan (5.8 µM), or their combination for 48 h. Following treatment, cells were incubated with JC-1 dye (10 µg/mL) in complete medium for 30 min at 37 °C in the dark.

Fluorescence was analyzed by flow cytometry using a FACSCalibur instrument (BD Biosciences, San Jose, CA, USA). A total of 10,000 events were acquired per sample. JC-1 aggregates (red fluorescence) and monomers (green fluorescence) were quantified, and the red/green fluorescence ratio was used as an indicator of mitochondrial membrane potential. All experiments were performed in triplicate and repeated in at least three independent experiments.

### 2.8. Immunocytochemical Staining

Immunocytochemical analysis was performed to evaluate caspase-9 expression. Y79 cells were seeded onto poly-L-lysine-coated coverslips at a density of 5 × 10^4^ cells per coverslip and allowed to stabilize for 24 h prior to treatment.

Cells were treated with quercetin (36.7 µM), topotecan (5.8 µM), or their combination for 48 h. Following treatment, cells were fixed with 4% paraformaldehyde for 15 min at room temperature, permeabilized with 0.1% Triton X-100 (Sigma-Aldrich, St. Louis, MO, USA)in PBS for 10 min, and blocked with 5% bovine serum albumin (BSA) in PBS for 1 h to reduce non-specific binding.

Cells were then incubated overnight at 4 °C with a primary rabbit anti-caspase-9 antibody (1:200 dilution). After washing with PBS, cells were incubated with an appropriate HRP-conjugated secondary antibody. Immunoreactivity was visualized using 3,3′-diaminobenzidine (DAB), resulting in brown staining. Nuclei were counterstained with hematoxylin.

Coverslips were mounted and examined under a light microscope. Caspase-9 expression was evaluated using semi-quantitative H-score analysis based on staining intensity (0 = negative, 1 = weak, 2 = moderate, 3 = strong) and the percentage of positive cells.

### 2.9. Gene Expression Analysis (qRT-PCR)

Total RNA was extracted from treated Y79 cells using TRIzol reagent (Invitrogen, Thermo Fisher Scientific, Waltham, MA, USA; 15596026) according to the manufacturer’s instructions. RNA concentration and purity were assessed spectrophotometrically using a NanoDrop instrument (Thermo Scientific, Waltham, MA, USA) by measuring the A260/A280 ratio.

Complementary DNA (cDNA) was synthesized from 1 µg of total RNA using the iScript cDNA Synthesis Kit (Bio-Rad, Hercules, CA, USA; 1708891). Quantitative real-time PCR (qRT-PCR) was performed using SYBR Green Master Mix (Bio-Rad, Hercules, CA, USA) on a real-time PCR system.

The expression levels of apoptosis-related genes (*CASP3*, *CASP9*, *BAX*, and *BCL2*) and *PI3K/Akt/mTOR* pathway components (*PIK3CA*, *AKT1*, *MTOR*, and *PTEN*) were analyzed, with *GAPDH* used as the internal reference gene. Primer sequences are provided in Table 1.

**Table 1:** Primer sequences used in qRT-PCR analysis.

Gene	Forward (5′ → 3′)	Reverse (5′ → 3′)
*CASP3*	AGAACTGGACTGTGGCATTGAG	GCTTGTCGGCATACTGTTTCAG
*CASP9*	CCTGACTTTGAGGACCTTCG	AGTCTGGCTCGGGGTTACTG
*BAX*	CCCGAGAGGTCTTTTTCCGAG	CCAGCCCATGATGGTTCTGAT
*BCL2*	GGTGGGGTCATGTGTGTGG	CGGTTCAGGTACTCAGTCATCC
*PIK3CA*	TGCTAAAGAAATCTTTCTCCTGG	TGGTGTGGAAGATCCAATCC
*AKT1*	ATGAGCGACGTGGCTATTGT	GAGGCCGTCAGCCACAGTCT
*MTOR*	CTTCTATGACCAACCCCAAGC	TCGATGTCTTGATCCAGGGT
*PTEN*	TGGATTCGACTTAGACTTGACCT	GGTGGGTTATGGTCTTCAAAAGG
*GAPDH*	GAAGGTGAAGGTCGGAGTC	GAAGATGGTGATGGGATTTC

All primer sequences are presented in the 5′ → 3′ direction. GAPDH was used as the internal reference gene.

Relative gene expression levels were calculated using the 2^−ΔΔCt^ method and normalized to untreated control samples. All reactions were performed in triplicate and repeated in at least three independent experiments.

### 2.10. Three-Dimensional Retinoblastoma Tumor Spheroid Experiments

#### 2.10.1. Establishment of 3D Tumor Spheroids

To more accurately model the architectural complexity, cell–cell interactions, and diffusion constraints of solid tumors, a three-dimensional (3D) spheroid model was established using Y79 retinoblastoma cells (ATCC^®^ HTB-18™).

Cells were cultured in RPMI-1640 medium supplemented with 20% FBS, 1% L-glutamine, and 1% penicillin–streptomycin under standard conditions (37 °C, 5% CO_2_). Cells in the logarithmic growth phase were harvested and seeded into ultra-low attachment round-bottom 96-well plates at a density of 3 × 10^3^ cells per well in 200 µL medium.

Ultra-low attachment conditions prevented surface adhesion and promoted spontaneous aggregation. Within 48–72 h of culture, compact, spherical spheroids with uniform morphology and smooth borders were formed. At the time of treatment initiation, spheroids exhibited a consistent morphology with an average diameter range of approximately 250–350 µm, ensuring experimental uniformity. Only structurally consistent spheroids were selected to minimize variability in downstream analyses.

#### 2.10.2. Treatment of 3D Tumor Spheroids

Following spheroid maturation, treatment was initiated by replacing 50% of the culture medium with fresh medium containing quercetin (36.7 µM), topotecan (5.8 µM), or their combination (1:2 ratio).

These concentrations were selected based on IC_50_ values obtained from 2D monolayer experiments and used as operational reference points rather than absolute biological thresholds. Considering the presence of diffusion barriers and slower drug penetration in 3D systems, spheroids were exposed to treatments for 72 h to allow adequate drug distribution and biological response. Vehicle controls (DMSO < 0.1%) were included.

#### 2.10.3. Bright-Field Imaging and Morphometric Analysis

Spheroid morphology was documented using inverted light microscopy under standardized imaging conditions. Both spheroid diameter and morphological integrity were used as quantitative and qualitative endpoints. Spheroid diameter was measured using ImageJ software (National Institutes of Health, Bethesda, MD, USA; version 1.53). For each spheroid, two orthogonal measurements were taken at the widest points, and the mean value was calculated. This approach minimized bias due to treatment-induced irregularity.

#### 2.10.4. ATP-Based Viability Assay

Cell viability was assessed using the CellTiter-Glo^®^ 3D assay (Promega Corporation, Madison, WI, USA), which quantifies intracellular ATP as a marker of metabolically active cells. This assay served as the primary quantitative metric for spheroid viability following treatment.

An equal volume of reagent was added to each well to ensure complete spheroid lysis. Plates were incubated with orbital shaking to facilitate ATP release. Luminescence was measured and normalized to control spheroids.

#### 2.10.5. Live/Dead Fluorescence Imaging

The spatial distribution of viable and non-viable cells was evaluated using Calcein-AM (live, green) and Ethidium homodimer-1 (dead, red) staining.

Fluorescence imaging was performed under identical acquisition parameters. No post-acquisition processing was applied.

### 2.11. Assessment of ROS-Associated Cytotoxicity by NAC Pretreatment

#### 2.11.1. NAC Pretreatment Strategy and Controls

Given the increased oxidative stress observed in 2D experiments, additional validation was performed in 3D spheroids using NAC pretreatment. To determine whether treatment-induced cytotoxicity is mediated by oxidative stress, antioxidant rescue experiments were performed using NAC.

Mature spheroids were pretreated with 5 mM NAC for 1 h at 37 °C prior to drug exposure. This concentration was selected based on its ability to effectively replenish intracellular glutathione levels and neutralize ROS without inducing cytotoxicity.

To rigorously control for NAC-related effects, the following experimental groups were included:

- Control (untreated);
- Quercetin;
- Topotecan;
- Combination;
- Combination + NAC.

Following pretreatment, NAC was maintained throughout the treatment period, ensuring continuous ROS suppression.

#### 2.11.2. Spheroid Dissociation and ROS Measurement

Due to diffusion gradients within spheroids, intracellular ROS was quantified at the single-cell level.

Following treatment, spheroids were enzymatically dissociated using Accutase^®^ (Innovative Cell Technologies, San Diego, CA, USA) to obtain homogeneous single-cell suspensions. Cells were washed with PBS and incubated with 10 µM DCFH-DA for 30 min at 37 °C in the dark.

Fluorescence intensity was measured at 485/528 nm, and ROS levels were expressed relative to the control.

#### 2.11.3. Functional Assessment of ROS Contribution

To evaluate whether ROS contributes to cytotoxicity, spheroid viability was assessed following NAC pretreatment using the CellTiter-Glo^®^ 3D assay (Promega Corporation, Madison, WI, USA).

An increase in viability in NAC-treated groups relative to non-NAC-treated groups was interpreted as supportive evidence for the contribution of ROS to treatment-induced cytotoxicity.

### 2.12. Bioinformatics Analyses

#### 2.12.1. Analysis of Gene Expression Datasets

To contextualize experimental findings with publicly available transcriptomic data, two Gene Expression Omnibus (GEO) datasets (GSE110811 and GSE97508), comprising retinoblastoma cell lines and healthy retinal samples, were retrieved from the GEO database (https://www.ncbi.nlm.nih.gov/geo/, accessed on 15 February 2026).

Data preprocessing, normalization, and differential expression analysis were performed using the GEO2R online analytical tool. Genes were considered differentially expressed if they met the criteria of |log_2_ fold change| ≥ 1 and an adjusted *p*-value < 0.05, based on the Benjamini–Hochberg false discovery rate (FDR) correction method.

#### 2.12.2. Protein–Protein Interaction Network Analysis

A protein–protein interaction (PPI) network was constructed to explore interactions among key components of the PI3K/Akt/mTOR signaling pathway. Interaction data were obtained using the STRING database (https://string-db.org/, accessed on 15 February 2026), and only interactions with a combined confidence score ≥ 0.7 were retained to ensure high-confidence associations.

The resulting network was imported into Cytoscape software (version 3.9.1) for visualization and topological analysis.

### 2.13. Statistical Analysis

All experiments were performed with three independent biological replicates. Data are presented as mean ± standard deviation (SD). Statistical comparisons between multiple groups were performed using one-way analysis of variance (ANOVA) followed by Tukey’s multiple comparison test. Within the bioinformatics analyses, differential gene expression was assessed using the limma moderated *t*-test with Benjamini–Hochberg false discovery rate (FDR) correction, and a significance threshold of FDR < 0.05 was applied. Pearson’s correlation coefficient was used to evaluate gene co-expression relationships. All statistical analyses were performed using GraphPad Prism 9.0 (GraphPad Software, San Diego, CA, USA). A *p* < 0.05 was considered statistically significant.

## 3. Results

### 3.1. Cell Viability and Cytotoxicity Findings

**Figure 1:**
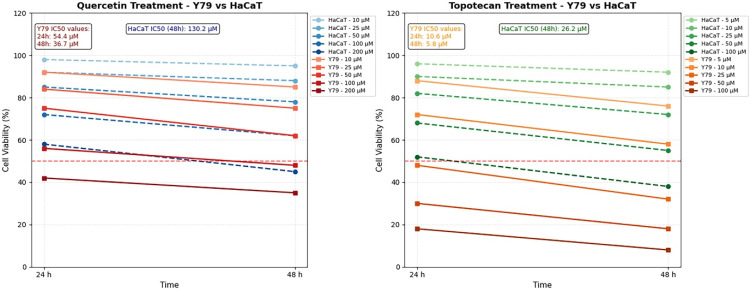
Cytotoxic effects of quercetin and topotecan on Y79 retinoblastoma and HaCaT keratinocyte cells. Cell viability was assessed using the MTT assay following treatment with increasing concentrations of quercetin (0–200 µM) and topotecan (0–100 µM) for 24 and 48 h. Both agents induced concentration- and time-dependent reductions in cell viability, with Y79 cells exhibiting greater sensitivity compared to HaCaT cells. IC_50_ values were calculated for each condition and are indicated within the graphs. Data are presented as mean ± SD from three independent biological replicates (*n* = 3). Statistical analysis was performed using one-way ANOVA followed by Tukey’s multiple comparison test.

MTT-based dose–response profiling revealed concentration- and time-dependent growth inhibition by both agents in Y79 and HaCaT cells. In Y79 retinoblastoma cells, quercetin yielded an IC_50_ of 54.4 µM at 24 h, which decreased to 36.7 µM at 48 h; the corresponding topotecan IC_50_ values were 10.6 µM and 5.8 µM at the two time points, respectively. HaCaT keratinocytes proved substantially less sensitive, with 48 h IC_50_ values of 130.2 µM for quercetin and 26.2 µM for topotecan, both of which were significantly higher than those in Y79 cells, indicating preferential cytotoxicity toward the malignant cell type. The 48 h IC_50_ concentrations in Y79 cells (quercetin 36.7 µM; topotecan 5.8 µM) were adopted as reference doses for all subsequent mechanistic experiments (Figure 1).

### 3.2. Combination Index and Synergy Analysis Findings

**Figure 2:**
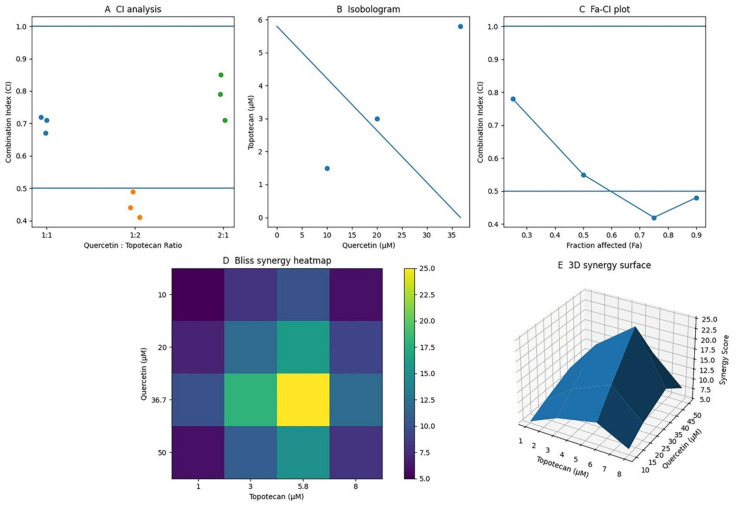
Integrated analysis of the synergistic interaction between quercetin and topotecan in Y79 retinoblastoma cells: (**A**) CI analysis for fixed-ratio combinations of quercetin and topotecan (1:1, 1:2, and 2:1). Data points represent calculated CI values, and horizontal lines indicate thresholds for synergism (CI < 1) and strong synergism (CI < 0.5). Different colored circles represent different quercetin: topotecan ratios. (**B**) Isobologram analysis based on the IC_50_ values of quercetin (36.7 µM) and topotecan (5.8 µM). The line connecting single-agent IC_50_ values represents the line of additivity, while combination data points correspond to experimentally derived doses; points located below the line indicate synergistic interaction. (**C**) Fraction-affected (Fa)–CI plot generated according to the Chou–Talalay method, illustrating drug interaction across different effect levels. (**D**) Bliss synergy heatmap depicting the interaction landscape across different concentration combinations, where warmer colors indicate higher synergy scores. (**E**) Three-dimensional synergy surface illustrating the distribution of Bliss synergy scores across the dose–response matrix. Collectively, these analyses are consistent with a synergistic interaction between quercetin and topotecan in Y79 cells.

Chou–Talalay CI analysis across three fixed molar ratios (1:1, 1:2, 2:1) consistently yielded CI values below 1.0, indicating synergistic interactions (CI < 1) across the tested conditions. The most pronounced effect was observed at the 1:2 quercetin-to-topotecan ratio, where the CI reached a minimum of approximately 0.42 at the paired doses of 36.7 µM quercetin and 5.8 µM topotecan, indicating strong synergism (CI < 0.5). CI values remained consistently below 1.0 across all concentrations, further supporting the robustness of the interaction. Based on these findings, the 36.7/5.8 µM (1:2) combination was selected for subsequent mechanistic analyses (Figure 2). The calculated CI values ranged from approximately 0.42 to 0.85 across the tested concentration ratios, indicating consistent synergistic interactions (CI < 1) under all experimental conditions.

### 3.3. Intracellular ROS Levels and ROS Suppression (Rescue) Experiment

DCFH-DA fluorimetric analysis at 48 h demonstrated a marked increase in intracellular ROS levels following treatment. Relative to untreated controls, quercetin (36.7 µM) increased ROS levels to approximately 157.0%, while topotecan (5.8 µM) elevated ROS to 186.9%. The combination treatment further amplified ROS accumulation to 250.6%, exceeding the levels observed with either monotherapy.

To evaluate the contribution of oxidative stress, an NAC rescue experiment was performed. Co-treatment with 5 mM NAC markedly reduced ROS levels in the combination group to 137.7%, indicating effective attenuation of treatment-induced oxidative stress.

**Figure 3:**
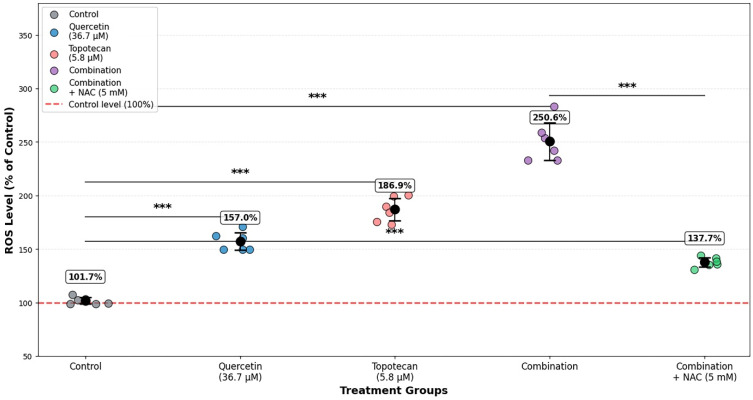
Intracellular ROS levels in Y79 cells following quercetin and topotecan treatment. Cells were treated with quercetin (36.7 µM), topotecan (5.8 µM), their combination, or combination plus NAC (5 mM) for 48 h. Intracellular ROS levels were measured using a DCFH-DA-based fluorometric assay and expressed as percentages relative to untreated control cells. Data are presented as mean ± SD from three independent biological replicates (*n* = 3). Statistical analysis was performed using one-way ANOVA followed by Tukey’s multiple comparison test (*** *p* < 0.001 vs. control).

Collectively, these findings indicate increased ROS levels in response to the quercetin–topotecan combination (Figure 3).

### 3.4. Apoptosis Analysis Findings (Annexin V-FITC/PI Staining)

Annexin V-FITC/PI dual staining with flow cytometric acquisition of 20,000 events per sample enabled the classification of cells into four distinct populations. In untreated controls, viable cells (Annexin V^−^/PI^−^) represented 94.7% of events. Single-agent exposure reduced this fraction to 60.5% with quercetin (36.7 µM) and to 40.5% with topotecan (5.8 µM), while combination treatment further decreased viability to 30.8%.

Early apoptotic cells (Annexin V^+^/PI^−^) increased from 2.4% in controls to 17.5%, 30.8%, and 31.6% in the quercetin, topotecan, and combination groups, respectively. Late apoptotic cells (Annexin V^+^/PI^+^) increased from 2.1% to 12.8%, 18.9%, and 22.7% across the same conditions.

When combined, total apoptotic fractions reached 4.5% in controls and increased to 30.3%, 49.7%, and 54.3% in the quercetin, topotecan, and combination groups, respectively (all *p* < 0.001). The combination treatment resulted in higher total apoptosis compared to both quercetin and topotecan monotherapies.

Necrotic cell proportions remained relatively low (control: 0.8%; quercetin: 9.2%; topotecan: 8.5%; combination: 10.1%), indicating that apoptosis was the predominant mode of cell death.

**Figure 4:**
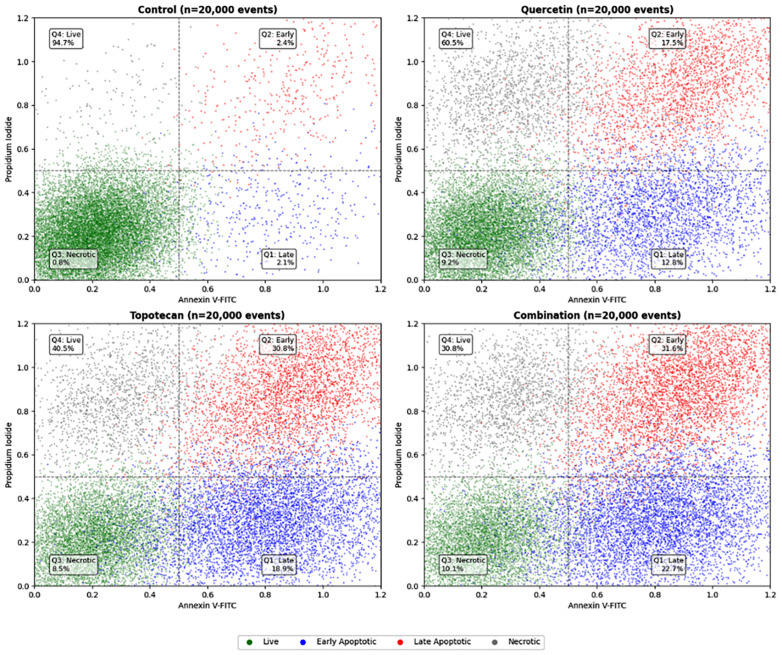
Annexin V-FITC/PI flow cytometric analysis of apoptosis in Y79 cells. Cells were treated with quercetin (36.7 µM), topotecan (5.8 µM), or their combination for 48 h. A total of 20,000 events were acquired per sample. Representative dot plots from three independent experiments are shown, displaying Annexin V-FITC (x-axis) and propidium iodide (PI; y-axis) fluorescence. Cell populations were classified as viable (Annexin V^−^/PI^−^), early apoptotic (Annexin V^+^/PI^−^), late apoptotic (Annexin V^+^/PI^+^), and necrotic (Annexin V^−^/PI^+^). The percentage of cells in each population is indicated. Quantitative analysis of apoptotic populations revealed that combination treatment increased both early and late apoptotic cell populations compared to single treatments (*p* < 0.001). Data are presented as mean ± SD from three independent biological replicates (*n* = 3). Statistical analysis was performed using one-way ANOVA followed by Tukey’s multiple comparison test.

Collectively, these findings indicate an enhanced pro-apoptotic effect of the quercetin–topotecan combination in Y79 cells, consistent with increased apoptotic cell death (Figure 4).

### 3.5. Changes in Mitochondrial Membrane Potential (ΔΨm)

JC-1 staining demonstrated treatment-dependent alterations in mitochondrial membrane potential (ΔΨm). In control cells, a predominant red fluorescence signal corresponding to JC-1 aggregates indicated intact mitochondrial polarization.

Quercetin and topotecan treatments resulted in a noticeable shift toward increased green fluorescence, reflecting partial mitochondrial depolarization. This shift was more pronounced in the combination group, where a substantial increase in green fluorescence and reduction in red signal were observed, indicating enhanced mitochondrial depolarization.

**Figure 5:**
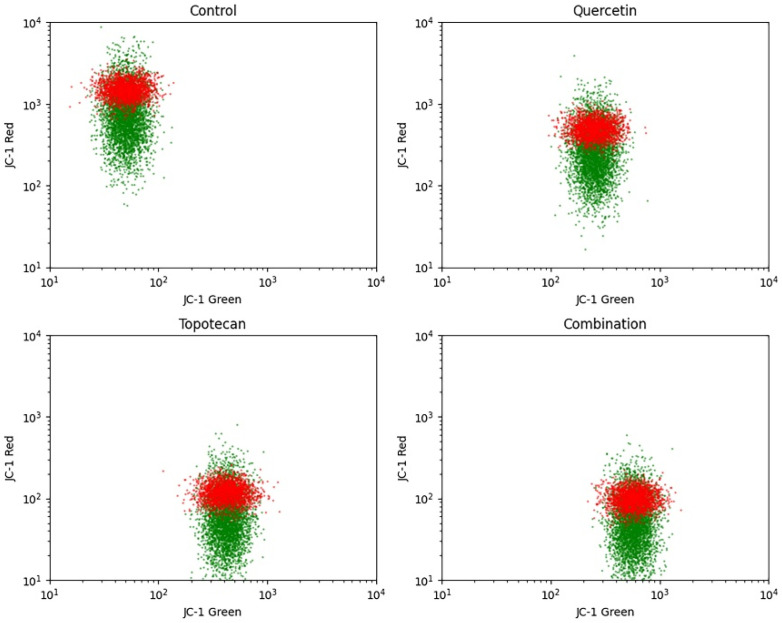
JC-1–based mitochondrial membrane potential (ΔΨm) analysis in Y79 cells. Cells were treated with quercetin (36.7 µM), topotecan (5.8 µM), or their combination for 48 h. A total of 10,000 events were acquired per sample by flow cytometry following JC-1 staining. Representative density plots from three independent experiments show red fluorescence (JC-1 aggregates, indicating polarized mitochondria) versus green fluorescence (JC-1 monomers, indicating depolarized mitochondria). The dashed line indicates the balance between red and green fluorescence signals. Control cells predominantly exhibited red fluorescence, whereas treated groups displayed a shift toward increased green fluorescence, most prominently in the combination group, consistent with enhanced mitochondrial depolarization. Quantitative analysis of mitochondrial membrane potential was performed using the ratio of red (JC-1 aggregates) to green (JC-1 monomers) fluorescence. Data are presented as mean ± SD from three independent biological replicates (*n* = 3). Statistical analysis was performed using one-way ANOVA followed by Tukey’s multiple comparison test.

These findings indicate greater disruption of mitochondrial membrane integrity compared to single-agent treatments (Figure 5).

### 3.6. Immunocytochemical Staining Findings

Immunocytochemical analysis of caspase-9 expression demonstrated a treatment-dependent increase in staining intensity in Y79 cells. Control cells exhibited weak and diffuse cytoplasmic immunoreactivity. Following 48 h of treatment, both quercetin (36.7 µM) and topotecan (5.8 µM) increased caspase-9 expression compared to the control, with a more pronounced effect observed in the combination group.

Quantitative H-score analysis confirmed this trend, showing a progressive increase in caspase-9 expression across treatment groups, with the highest levels detected in the combination-treated cells (*p* < 0.001 vs. control).

**Figure 6:**
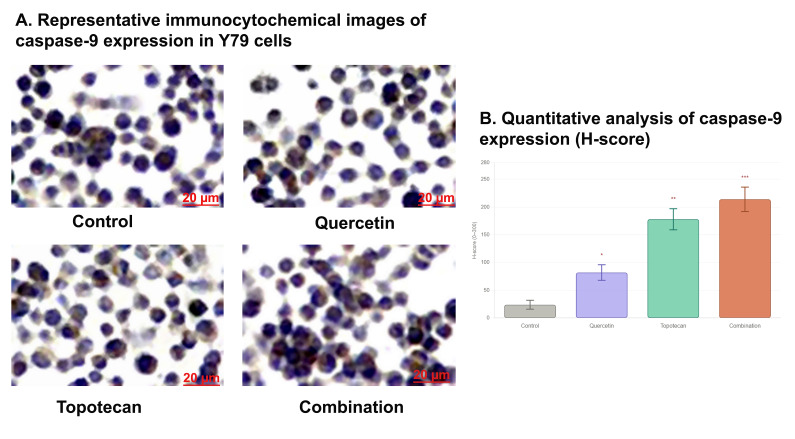
Caspase-9 expression in Y79 cells treated with quercetin, topotecan, and their combination as determined by immunocytochemistry: (**A**) Brown (DAB) staining indicates caspase-9 immunoreactivity, while hematoxylin counterstaining (blue–purple) marks cell nuclei. Scale bar: 20 µm. (**B**) Quantitative analysis of caspase-9 expression based on H-score evaluation. H-score values were calculated as the sum of the percentage of positive cells multiplied by staining intensity (0 = negative, 1 = weak, 2 = moderate, 3 = strong). Data are presented as mean ± SD from three independent biological replicates (*n* = 3). Statistical analysis was performed using one-way ANOVA followed by Tukey’s multiple comparison test (* *p* < 0.05, ** *p* < 0.01, *** *p* < 0.001 vs. control).

These findings indicate enhanced activation of apoptotic signaling in response to combination treatment (Figure 6).

### 3.7. Gene Expression Analysis Findings (qRT-PCR)

qRT-PCR analysis revealed that the quercetin–topotecan combination induced significant changes in the expression of both apoptotic and survival-related genes in Y79 cells. Among pro-apoptotic transcripts, *CASP3* was upregulated to 5.0 ± 0.4 fold (*p* < 0.001), *CASP9* to 5.8 ± 0.5 fold (*p* < 0.001), and *BAX* to 4.4 ± 0.4 fold (*p* < 0.001). In contrast, anti-apoptotic *BCL2* expression was reduced to 0.28 ± 0.04 fold relative to the control (*p* < 0.001).

**Figure 7:**
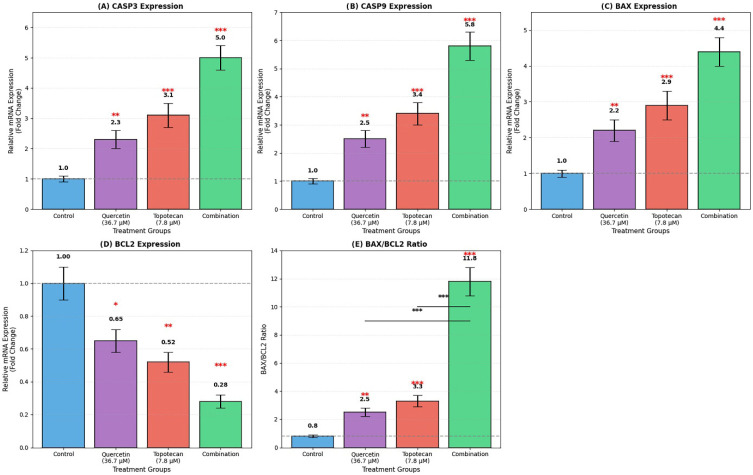
Apoptosis-related gene expression analysis in Y79 cells by qRT-PCR. Cells were treated with quercetin, topotecan, and their combination for 48 h. mRNA expression levels of (**A**) *CASP3*, (**B**) *CASP9*, (**C**) *BAX*, and (**D**) *BCL2* were determined by qRT-PCR. (**E**) The *BAX/BCL2* ratio was calculated from relative expression levels. Gene expression was normalized to *GAPDH* and expressed as fold change relative to untreated control cells. The combination treatment increased pro-apoptotic gene expression (*CASP3*: 5.0 fold, *CASP9*: 5.8 fold, and *BAX*: 4.4 fold) while reducing anti-apoptotic *BCL2* expression (0.28 fold), resulting in an elevated *BAX/BCL2* ratio (11.8 ± 1.0). Data are presented as mean ± SD from three independent biological replicates (*n* = 3). Statistical analysis was performed using one-way ANOVA followed by Tukey’s multiple comparison test (* *p* < 0.05, ** *p* < 0.01, *** *p* < 0.001 vs. control).

The resulting BAX/BCL2 ratio increased from 0.8 ± 0.1 in untreated cells to 2.5 ± 0.3 with quercetin, 3.3 ± 0.4 with topotecan, and 11.8 ± 1.0 under combination treatment (*p* < 0.001 for all comparisons), suggesting a shift toward a pro-apoptotic gene expression pattern (Figure 7).

**Figure 8:**
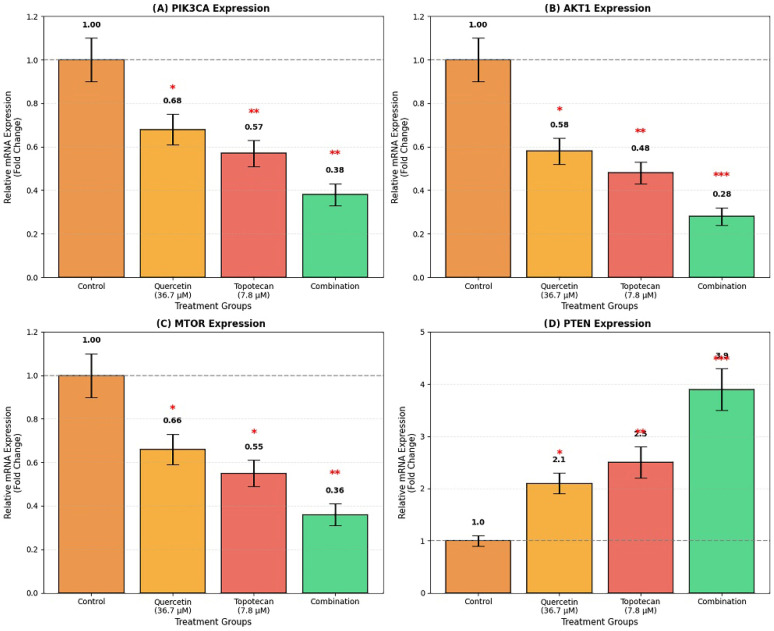
PI3K/Akt/mTOR pathway gene expression analysis in Y79 cells by qRT-PCR. Cells were treated with quercetin, topotecan, and their combination for 48 h. mRNA expression levels of (**A**) *PIK3CA*, (**B**) *AKT1*, (**C**) *MTOR*, and (**D**) *PTEN* were determined by qRT-PCR. Gene expression was normalized to *GAPDH* and expressed as fold change relative to untreated control cells. The combination treatment reduced *PIK3CA* (0.38 fold), *AKT1* (0.28 fold), and *MTOR* (0.36 fold) expression while increasing *PTEN* expression (3.9 fold). Single treatments showed similar but less pronounced effects. Data are presented as mean ± SD from three independent biological replicates (*n* = 3). Statistical analysis was performed using one-way ANOVA followed by Tukey’s multiple comparison test (* *p* < 0.05, ** *p* < 0.01, *** *p* < 0.001 vs. control).

**Figure 9:**
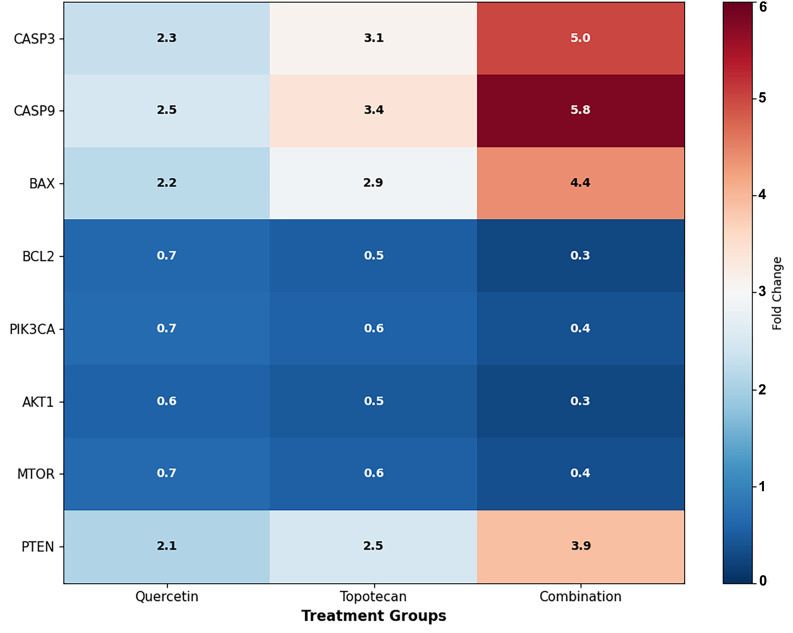
Summary heatmap of gene expression changes. Heatmap illustrating fold changes in apoptosis-related genes (*CASP3*, *CASP9*, *BAX*, and *BCL2*) and PI3K/Akt/mTOR pathway genes (*PIK3CA*, *AKT1*, *MTOR*, and *PTEN*) following quercetin, topotecan, and combination treatments. Red indicates upregulation, whereas blue indicates downregulation relative to the control. The combination treatment shows more pronounced changes across the analyzed genes, indicating enhanced modulation of both apoptotic and survival-related pathways.

Parallel analysis of PI3K/Akt/mTOR pathway transcripts demonstrated that combination treatment coordinately downregulated key pathway components, including *PIK3CA* (0.38 ± 0.05 fold; *p* < 0.01), *AKT1* (0.28 ± 0.04 fold; *p* < 0.001), and *MTOR* (0.36 ± 0.05 fold; *p* < 0.01). Conversely, the tumor suppressor *PTEN* was upregulated to 3.9 ± 0.4 fold (*p* < 0.001). Single-agent treatments induced directionally similar but less pronounced effects: quercetin reduced *PIK3CA*, *AKT1*, and *MTOR* expression to 0.68 ± 0.07, 0.58 ± 0.06, and 0.66 ± 0.07 fold, respectively, while increasing *PTEN* expression to 2.1 ± 0.2 fold. Similarly, topotecan decreased *PIK3CA* (0.57 ± 0.06 fold), *AKT1* (0.48 ± 0.05 fold), and *MTOR* (0.55 ± 0.06 fold) while elevating *PTEN* expression to 2.5 ± 0.3 fold. Overall, the combination treatment produced the most pronounced modulation of PI3K/Akt/mTOR pathway-related gene expression (Figure 8 and Figure 9).

### 3.8. Effects of Topotecan and Quercetin in 3D Tumor Spheroids

To validate the findings obtained from 2D monolayer experiments under more physiologically relevant conditions, 3D tumor spheroid experiments were performed.

#### 3.8.1. Morphological Alterations

**Figure 10:**
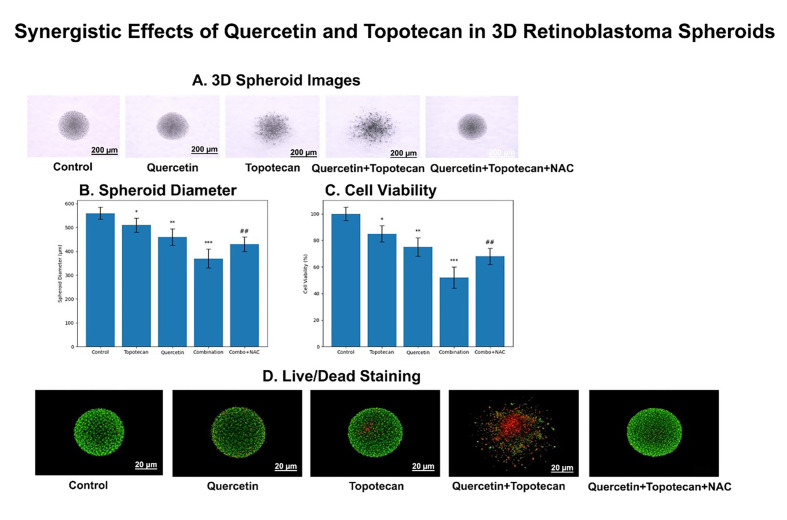
Synergistic effects of quercetin and topotecan in 3D retinoblastoma spheroids: (**A**) Representative bright-field images of Y79 spheroids following treatment with quercetin (36.7 µM), topotecan (5.8 µM), their combination, and combination plus NAC (5 mM). Control spheroids exhibited compact, spherical morphology with smooth borders, whereas treated groups showed progressive structural disruption, most prominently in the combination group. NAC co-treatment partially preserved spheroid integrity. Scale bar: 200 µm. (**B**) Quantitative analysis of spheroid diameter. All treatments significantly reduced spheroid size compared to the control, with the combination group showing the most pronounced decrease. NAC co-treatment partially restored spheroid size relative to the combination group. (**C**) ATP-based viability assay of 3D spheroids. All treatment groups exhibited reduced viability, with the greatest reduction observed in the combination group. NAC co-treatment partially increased cell viability compared to the combination treatment alone. (**D**) Live/Dead fluorescence staining using Calcein-AM (green, viable cells) and Ethidium homodimer-1 (red, dead cells). Control spheroids displayed predominantly viable cells, whereas combination treatment induced extensive cell death and structural disintegration. NAC co-treatment partially reduced cell death. These representative images visually corroborate the quantitative findings obtained from spheroid size and ATP-based viability analyses. Scale bar: 20 µm. Data are presented as mean ± SD from three independent biological replicates (*n* = 3). Statistical analysis was performed using one-way ANOVA followed by Tukey’s multiple comparison test (* *p* < 0.05, ** *p* < 0.01, *** *p* < 0.001 vs. control; ## *p* < 0.01 vs. combination group).

Control spheroids exhibited compact, spherical morphology with smooth borders (Figure 10A). Quercetin treatment resulted in moderate size reduction with preserved structure. Topotecan caused more pronounced shrinkage and partial loss of compactness. The combination treatment induced severe structural disruption, including irregular borders, reduced density, and fragmentation, indicating enhanced cytotoxicity (Figure 10A).

#### 3.8.2. Spheroid Size Reduction

Quantitative analysis confirmed a statistically significant reduction in spheroid diameter across all treatment groups, with the combination group showing the most pronounced decrease compared to single treatments (Figure 10B) (*p* < 0.001).

#### 3.8.3. Decreased Viability

ATP-based analysis demonstrated a significant decrease in spheroid viability in all treatment groups, with the combination treatment showing the greatest reduction, consistent with synergistic cytotoxicity (Figure 10C) (*p* < 0.001).

#### 3.8.4. Live/Dead Fluorescence

Control spheroids displayed predominantly green fluorescence (Figure 10D). Single treatments increased the red signal. The combination group exhibited dominant red fluorescence, indicating widespread cell death and structural collapse (Figure 10D).

Representative bright-field and fluorescence images illustrating treatment-induced structural disruption and cell death are shown in Figure 10, supporting both morphological and viability-related findings. While Live/Dead fluorescence imaging provided qualitative evidence of treatment-induced cell death, quantitative analysis of red fluorescence intensity or the proportion of dead cells across spheroid regions was not performed in the present study. Future studies incorporating image-based quantification and correlation with ATP-based viability assays would further strengthen the interpretation of these findings.

### 3.9. NAC Pretreatment Suggests ROS Contribution in 3D Spheroids

#### 3.9.1. ROS Induction

**Figure 11:**
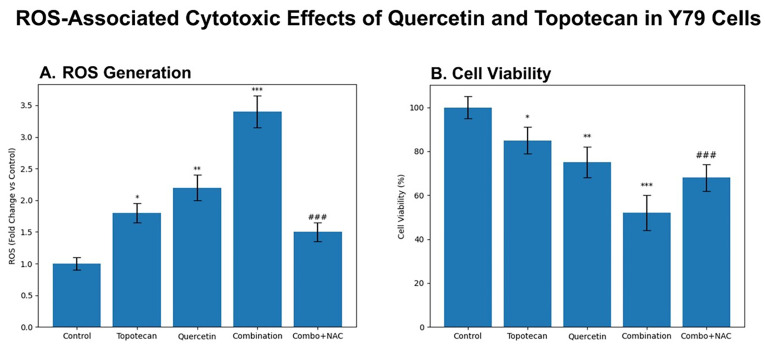
ROS-associated cytotoxic effects of quercetin and topotecan in Y79 cells: (**A**) Intracellular ROS levels measured by DCFH-DA fluorescence following treatment with quercetin (36.7 µM), topotecan (5.8 µM), their combination, and combination plus NAC (5 mM). Both single treatments increased ROS levels compared to the control, while the combination treatment resulted in the highest ROS production. NAC co-treatment significantly attenuated ROS levels relative to the combination group. (**B**) Cell viability following identical treatments. Both quercetin and topotecan reduced cell viability, with the combination treatment producing the most pronounced decrease. NAC co-treatment partially restored cell viability compared to the combination group, although levels remained below the control level. Data are presented as mean ± SD from three independent biological replicates (*n* = 3). Statistical analysis was performed using one-way ANOVA followed by Tukey’s multiple comparison test (* *p* < 0.05, ** *p* < 0.01, *** *p* < 0.001 vs. control; ### *p* < 0.001 vs. combination group).

Topotecan significantly increased intracellular ROS levels, while quercetin induced a moderate elevation. The combination treatment resulted in the highest ROS production, consistent with increased oxidative stress. NAC pretreatment markedly reduced ROS levels compared to the combination group (Figure 11A).

**Figure 12:**
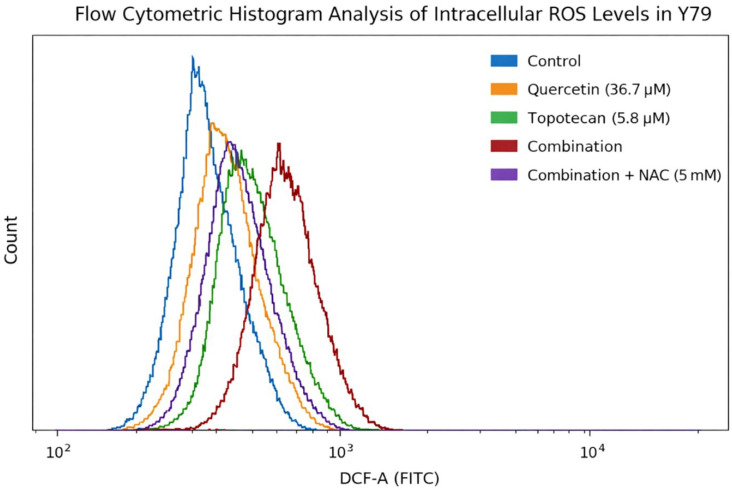
Flow cytometric histogram analysis of intracellular ROS levels in Y79 cells. Representative flow cytometric histograms of intracellular ROS levels measured using the DCFH-DA probe in Y79 cells following treatment with quercetin (36.7 µM), topotecan (5.8 µM), their combination, and combination plus NAC (5 mM). The combination treatment induced a pronounced rightward shift in fluorescence intensity compared to control and single-agent treatments, indicating increased intracellular ROS accumulation. Quercetin and topotecan alone produced moderate rightward shifts, consistent with partial ROS induction.

Flow cytometric histogram analysis further supported these findings, showing a pronounced rightward shift in DCF fluorescence intensity in the combination group compared with control and single-agent treatments (Figure 12). This shift reflects an overall increase in intracellular ROS distribution at the single-cell level. NAC pretreatment partially reversed this effect, as evidenced by a leftward shift in fluorescence intensity. However, as an NAC-only control group was not included in the current experimental design, potential independent effects of NAC on basal ROS levels cannot be fully excluded.

NAC pretreatment partially reversed the fluorescence shift toward control levels, reflecting attenuation of ROS generation. The distributions represent single-cell ROS measurements, highlighting treatment-dependent changes in intracellular oxidative stress.

#### 3.9.2. Partial Rescue of Cytotoxicity

NAC pretreatment significantly increased spheroid viability in the combination group (Figure 11B). However, viability remained below control levels, indicating an incomplete rescue effect.

#### 3.9.3. Mechanistic Interpretation

The incomplete recovery of viability despite ROS suppression suggests that ROS contributes to, but does not fully account for, the observed cytotoxicity. These findings support a multifactorial mechanism involving ROS-associated oxidative stress, mitochondrial dysfunction, and apoptotic signaling pathways. Accordingly, ROS appears to contribute to the cytotoxic response but is unlikely to be the sole driver of the underlying mechanism. Therefore, while the observed rescue effect is consistent with ROS involvement, it should be interpreted with caution in the absence of NAC-only controls.

### 3.10. Differential Gene Expression Analysis

Interrogation of the GEO transcriptome datasets GSE110811 and GSE97508 revealed expression patterns consistent with the experimental findings, showing upregulation of PI3K/Akt/mTOR pathway components in retinoblastoma compared to normal retina. Specifically, *PIK3CA* (log2FC = 2.34; *p* < 0.001), *AKT1* (log2FC = 1.87; *p* < 0.01), and *MTOR* (log2FC = 1.56; *p* < 0.01) were overexpressed in tumor samples, whereas the tumor suppressor *PTEN* was markedly downregulated (log2FC = −1.78; *p* < 0.01).

**Figure 13:**
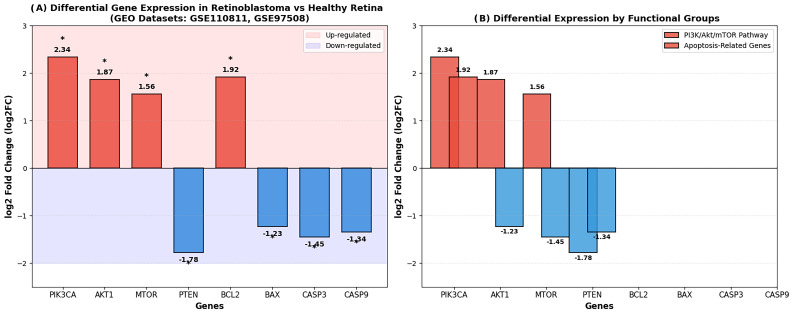
Differential gene expression analysis in retinoblastoma compared to healthy retinal tissue. Data were obtained from GEO datasets GSE110811 and GSE97508: (**A**) Bar chart showing log2 fold change (log2FC) of the PI3K/Akt/mTOR pathway and apoptosis-related genes in retinoblastoma samples relative to healthy retina. Positive values indicate upregulation, whereas negative values indicate downregulation in retinoblastoma. (**B**) Grouped bar chart illustrating differential expression by functional categories. PI3K/Akt/mTOR pathway genes (*PIK3CA*, *AKT1*, and *MTOR*) show upregulation, while the tumor suppressor *PTEN* is downregulated. Among apoptosis-related genes, the anti-apoptotic gene BCL2 is upregulated, whereas pro-apoptotic genes (*BAX*, *CASP3*, and *CASP9*) are downregulated. * *p* < 0.05 vs. control.

The apoptotic gene expression profile showed a similar trend: *BCL2* was upregulated (log2FC = 1.92; *p* < 0.01), while pro-apoptotic genes *BAX* (log2FC = −1.23; *p* < 0.05), *CASP3* (log2FC = −1.45; *p* < 0.05), and *CASP9* (log2FC = −1.34; *p* < 0.05) were downregulated in retinoblastoma tissue (Figure 13).

### 3.11. Protein–Protein Interaction Network and Correlation Analyses

The STRING-based PPI network illustrated high-confidence physical and functional associations among PIK3CA, AKT1, MTOR, PTEN, CASP3, CASP9, BAX, and BCL2, with both intra-pathway interactions within the PI3K/Akt/mTOR module and cross-talk connections linking these components to apoptotic regulators.

Pearson correlation analysis of gene expression data revealed coordinated expression patterns within these networks. *PIK3CA*, *AKT1*, and *MTOR* showed strong positive correlations (r > 0.7), whereas *PTEN* exhibited inverse correlations with these oncogenic components (r < −0.6). *BCL2* expression was positively associated with the PI3K/Akt/mTOR cluster (r > 0.6) and inversely correlated with pro-apoptotic genes *BAX*, *CASP3*, and *CASP9* (r < −0.5).

**Figure 14:**
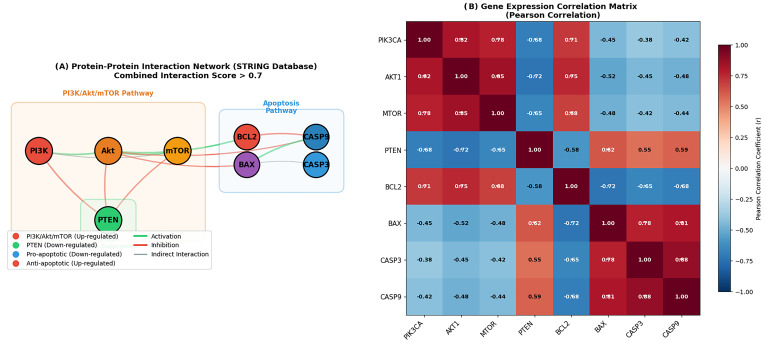
Protein–protein interaction network and gene expression correlation analysis: (**A**) Protein–protein interaction network constructed using the STRING database (combined interaction score > 0.7). Nodes represent proteins encoded by genes involved in the PI3K/Akt/mTOR pathway and apoptosis regulation. Node colors indicate expression patterns in retinoblastoma: red for upregulated genes (*PIK3CA*, *AKT1*, *MTOR*, and *BCL2*), green for the downregulated tumor suppressor *PTEN*, and blue for downregulated pro-apoptotic genes (*CASP3*, *CASP9*, and *BAX*). Edges represent predicted functional associations, with edge thickness reflecting interaction confidence. The network illustrates intra-pathway connectivity within PI3K/Akt/mTOR components and cross-talk with apoptotic regulators. (**B**) Heatmap showing Pearson correlation coefficients between gene expression levels. Red indicates positive correlation, and blue indicates negative correlation. Strong correlations (|r| > 0.6) are highlighted. *PIK3CA*, *AKT1*, and *MTOR* display strong positive correlations among themselves (r > 0.7), whereas *PTEN* shows negative correlations with these pathway components (r < −0.6). *BCL2* is positively associated with survival-related genes and negatively correlated with pro-apoptotic genes (*BAX*, *CASP3*, and *CASP9*).

These findings are consistent with a coordinated regulatory relationship between survival signaling and apoptotic pathways in retinoblastoma (Figure 14).

## 4. Discussion

Chemotherapy resistance remains a major challenge in advanced retinoblastoma, necessitating the development of combination strategies that may enhance cytotoxic efficacy while also overcoming adaptive survival mechanisms. In this preclinical study, we observed that the combination was associated with enhanced cytotoxic effects in Y79 retinoblastoma cells, consistent with synergistic interaction profiles. The observed synergistic interaction was consistently supported by CI analysis and further validated across complementary experimental platforms, including 2D monolayer systems and 3D spheroid models that more closely recapitulate tumor architecture and drug response. Importantly, the incorporation of NAC-mediated rescue experiments suggests that oxidative stress contributes to, but does not fully account for, the observed cytotoxic mechanism, supporting a multifactorial mode of action. This integrated response involves ROS-associated cellular stress, mitochondrial dysfunction, and modulation of apoptotic signaling pathways, alongside alterations in PI3K/Akt/mTOR-related survival signaling. The preferential sensitivity of Y79 cells relative to non-malignant HaCaT keratinocytes further supports a degree of tumor selectivity within this experimental context. These findings are consistent with previous reports identifying quercetin as a modulator of apoptosis and redox homeostasis across cancer models [16,20,21], while topotecan induces DNA damage-mediated cell death through replication stress and caspase activation [4]; however, our results extend this knowledge by suggesting that their combination is associated with coordinated and mechanistically integrated effects across multiple biological levels.

CI calculation via the Chou–Talalay method provided quantitative confirmation of synergism across all tested molar ratios, with the 1:2 quercetin-to-topotecan combination achieving a minimum CI of 0.42, well within the strong synergism threshold of CI < 0.5. Independent validation through isobologram and Bliss synergy surface analyses yielded concordant results. The emergence of synergism is consistent with the capacity of flavonoids to simultaneously target multiple oncogenic signaling nodes, thereby sensitizing cancer cells to cytotoxic agents through mechanisms that exceed simple dose addition [22,23].

A particularly noteworthy mechanistic finding was the dramatic amplification of intracellular ROS by the drug combination, which surpassed the oxidative burden produced by either agent individually. The ability of NAC pre-treatment to attenuate this response and partially rescue cell viability supports a contributory role for oxidative stress as a mechanistic contributor to synergistic cytotoxicity. Excessive ROS accumulation is known to impair mitochondrial membrane integrity and engage the intrinsic apoptotic machinery [24], and high oxidant loads within cancer cells are recognized as a pivotal trigger for caspase cascade activation [25].

Flow cytometric apoptosis profiling revealed that the combined regimen raised total programmed cell death to above 53% of the Y79 population, a level far exceeding either monotherapy, while necrotic fractions remained negligible across all conditions. The preponderance of Annexin V-positive over PI-positive cells confirms that the cytotoxic response is principally orchestrated through regulated apoptotic pathways rather than non-specific membrane disruption. This pattern is in line with prior reports demonstrating that flavonoid chemotherapeutic co-treatments preferentially amplify programmed cell death over necrosis [26].

JC-1 staining provided complementary evidence of mitochondrial dysfunction, with the red/green fluorescence ratio collapsing to 0.89 ± 0.07 under combination treatment, a roughly three-fold reduction relative to controls. Loss of the electrochemical gradient across the inner mitochondrial membrane is commonly associated with activation of the intrinsic apoptotic pathway, as it is known to be associated with cytochrome c release into the cytosol and subsequent apoptosome-mediated caspase activation [27]. The marked increase in caspase-9 immunoreactivity observed by immunocytochemical staining further supports this mechanistic interpretation. Furthermore, DAPI-based nuclear morphology assessment revealed appreciably higher rates of chromatin condensation and nuclear fragmentation in combination-treated cells relative to either monotherapy, further substantiating caspase-9-mediated execution of apoptosis through the mitochondrial route [28].

**Figure 15:**
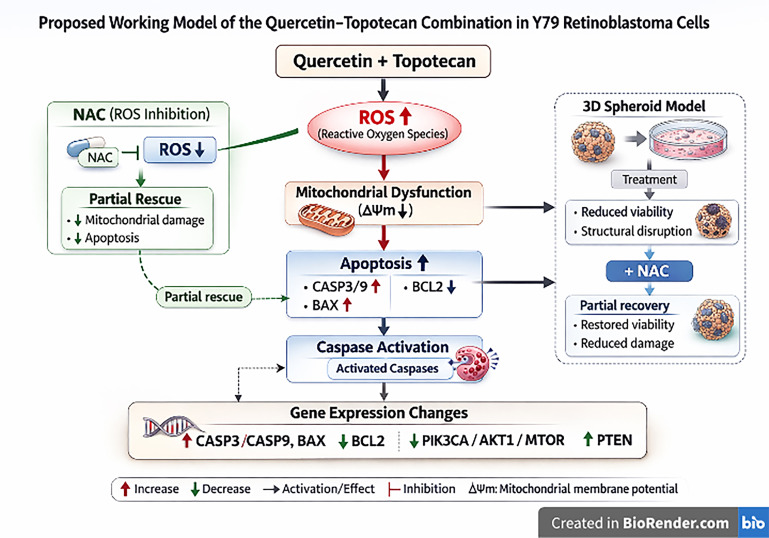
Proposed working model of the quercetin–topotecan combination in Y79 retinoblastoma cells. The schematic integrates the principal experimental findings of the study. The combination treatment is associated with increased intracellular ROS levels, mitochondrial membrane depolarization (ΔΨm), and enhanced apoptotic cell death accompanied by caspase activation. NAC-mediated attenuation of ROS levels and partial recovery of viability support a contributory role of oxidative stress. Observations from 3D spheroid models further indicate reduced viability and structural disruption, with partial recovery upon NAC treatment. Gene expression analyses demonstrate upregulation of pro-apoptotic markers (*CASP3*, *CASP9*, and *BAX*) and *PTEN*, alongside downregulation of *BCL2* and PI3K/Akt/mTOR-related transcripts. Arrows represent observed associations and do not imply direct causal relationships.

To provide an integrated overview of the observed findings, a conceptual working model is presented (Figure 15). This model summarizes the coordinated responses induced by the quercetin–topotecan combination, linking increased intracellular ROS levels with mitochondrial membrane depolarization and subsequent apoptotic cell death. The partial attenuation of these effects by NAC supports the functional involvement of oxidative stress while also indicating that additional mechanisms contribute to the overall cytotoxic response. Notably, the incorporation of 3D spheroid data into this framework highlights that these effects are not restricted to conventional monolayer systems but are also preserved under conditions that better approximate tumor architecture. Furthermore, the observed gene expression changes are included in the model to reflect the transcriptional landscape associated with the treatment response, without implying direct causal relationships. Collectively, this schematic representation is intended to contextualize the experimental findings within a multifactorial and integrative framework.

Gene expression profiling by qRT-PCR provided molecular-level resolution of the apoptotic and survival signaling changes underlying the observed phenotype. The combination upregulated *CASP3*, *CASP9*, and *BAX* while concurrently suppressing *BCL2*, yielding an 11.8-fold elevation of the *BAX/BCL2* ratio, a widely used transcriptional indicator of apoptotic commitment [29]. These changes collectively reinforce the conclusion that the combination engages the mitochondrial death pathway. Beyond the apoptotic gene set, the present data also revealed significant modulation of the PI3K/Akt/mTOR axis, a signaling hub governing proliferation, metabolic adaptation, and cell survival that is constitutively activated in numerous cancer types [30]. Combined quercetin–topotecan treatment reduced *PIK3CA*, *AKT1*, and *MTOR* transcript levels while reciprocally increasing *PTEN*, suggesting suppression of key pro-survival signaling components in Y79 cells. These findings are consistent with earlier reports linking hyperactivation of the PI3K/Akt/mTOR cascade to retinoblastoma progression and resistance [31].

The transcriptomic landscape of publicly available retinoblastoma datasets independently corroborated our experimental observations. GEO-derived expression profiles revealed coordinated upregulation of PI3K/Akt/mTOR effector genes alongside downregulation of pro-apoptotic transcripts in tumor versus normal retinal tissue, highlighting the clinical relevance of this signaling axis in retinoblastoma biology. STRING-based network modeling complemented these findings by mapping high-confidence physical interactions between PI3K/Akt/mTOR components and apoptotic proteins, revealing an intricate molecular dialogue between pro-survival and death-inducing networks [32]. The convergence of cell-based experimental data, transcriptomic evidence, and network topology analyses collectively strengthens the rationale for targeting the PI3K/Akt/mTOR pathway in retinoblastoma. Taken together, the results presented here suggest that quercetin is associated with enhanced apoptotic responses in retinoblastoma cells, accompanied by increased oxidative stress, mitochondrial dysfunction, and alterations in survival-related signaling components, which may contribute to the observed cytotoxic effects.

Several limitations inherent to the study design should be considered when interpreting these findings. Y79 cells were selected as a well-established RB1-deficient retinoblastoma model widely used in preclinical studies. However, the use of a single retinoblastoma cell line (Y79) limits the ability to capture the molecular heterogeneity of the disease. Retinoblastoma exhibits biological and molecular variability across different cell models; therefore, findings derived from a single cell line may not fully represent the spectrum of tumor responses. Validation of the present results in additional retinoblastoma cell lines, such as WERI-Rb1, would be important to confirm the broader applicability and robustness of the observed effects.

Although 3D spheroid models were incorporated to better approximate in vivo tumor architecture, these systems do not fully recapitulate the complexity of tumor microenvironmental interactions. Furthermore, the exclusively in vitro nature of the study precludes evaluation of pharmacokinetics, bioavailability, and systemic toxicity, which are essential for the translational application of the quercetin–topotecan combination.

At the mechanistic level, gene expression analyses were primarily performed at the transcript level; therefore, functional validation at the protein and post-translational levels remains limited. In particular, key signaling pathways such as PI3K/Akt/mTOR are predominantly regulated through phosphorylation-dependent mechanisms that cannot be fully captured by mRNA-based analyses alone. Future studies incorporating protein-level approaches, including Western blot analysis of cleaved caspase-3, Bax/Bcl-2 ratio, and phosphorylated Akt and mTOR (p-Akt, p-mTOR), would be necessary to confirm pathway modulation and strengthen mechanistic interpretation.

In particular, the observed upregulation of *PTEN* and downregulation of *PIK3CA*, *AKT1*, and *MTOR* transcripts in the present study warrant confirmation at the protein level, especially given the central role of phosphorylation-dependent activation in this pathway. While immunocytochemical analysis supported caspase-9 activation, broader pathway-level protein validation remains to be established. In addition, NAC-based rescue experiments indicated that oxidative stress contributes to the observed cytotoxicity; however, the incomplete rescue suggests that ROS is not the sole driver, supporting a multifactorial mechanism of action.

Future studies should validate these findings across additional retinoblastoma models and extend them to in vivo systems, including orthotopic tumor models, to assess therapeutic efficacy and safety in a physiological context.

Taken together, the present study provides preclinical evidence suggesting that the quercetin–topotecan combination is associated with enhanced antitumor activity through coordinated modulation of oxidative stress, mitochondrial dysfunction, apoptotic signaling, and PI3K/Akt/mTOR pathway components, warranting further investigation in translational settings.

From a translational perspective, the clinical application of the quercetin–topotecan combination presents several challenges. Quercetin is known to have limited bioavailability due to poor solubility and rapid metabolism, which may restrict its systemic efficacy. In contrast, topotecan is commonly administered via localized delivery routes in retinoblastoma, including intra-arterial and intravitreal administration, allowing high intraocular drug concentrations with reduced systemic exposure.

Future strategies may involve the development of localized delivery systems, such as nanoparticle-based formulations, liposomal carriers, or sustained-release intraocular platforms, to enhance quercetin bioavailability and enable co-administration with topotecan. Such approaches may improve intraocular drug retention and therapeutic efficacy while minimizing systemic toxicity. However, pharmacokinetic and formulation studies are required to determine the feasibility, stability, and optimal dosing of this combination in intraocular settings.

## 5. Conclusions

Collectively, the present study provides preclinical evidence suggesting that quercetin is associated with enhanced topotecan-induced cytotoxic effects in Y79 retinoblastoma cells under both two-dimensional (2D) and three-dimensional (3D) experimental conditions. These effects are supported by complementary findings, including increased apoptotic cell death, elevated intracellular ROS levels, and disruption of mitochondrial membrane potential. Importantly, validation in 3D tumor spheroid models, which partially recapitulate tumor architecture, further supports the biological relevance of these observations.

NAC-mediated rescue experiments indicate that oxidative stress contributes to, but does not fully account for, the observed cytotoxicity, consistent with a multifactorial mechanism involving ROS-associated cellular stress, mitochondrial dysfunction, and modulation of apoptotic and survival-related signaling pathways.

At the molecular level, the combination treatment was associated with upregulation of pro-apoptotic genes and downregulation of survival-related genes, alongside increased *PTEN* expression, suggesting a shift toward a pro-apoptotic gene expression profile.

These findings should be interpreted within the limitations of an in vitro preclinical model. Overall, quercetin may represent a potential adjunct strategy to modulate topotecan response; however, further validation in additional experimental systems and in vivo models is required before any translational or clinical implications can be considered.

## Data Availability

The original contributions presented in this study are included in the article. Further inquiries can be directed to the corresponding authors.

## Abbreviations

Akt	Protein kinase B
ANOVA	Analysis of variance
ATP	Adenosine triphosphate
BAX	Bcl-2-associated X protein
BCL2	B-cell lymphoma 2
CI	Combination index
DCFH-DA	2′:7′-Dichlorodihydrofluorescein diacetate
DMEM	Dulbecco’s Modified Eagle Medium
DMSO	Dimethyl sulfoxide
FBS	Fetal bovine serum
GAPDH	Glyceraldehyde-3-phosphate dehydrogenase
GEO	Gene Expression Omnibus
IC_50_	Half maximal inhibitory concentration
JC-1	5,5′,6,6′-Tetrachloro-1,1′,3,3′-tetraethylbenzimidazolylcarbocyanine iodide
MAPK	Mitogen-activated protein kinase
MTOR	Mechanistic target of rapamycin
NAC	N-acetyl-L-cysteine
PBS	Phosphate-buffered saline
PI	Propidium iodide
PI3K	Phosphoinositide 3-kinase
PIK3CA	Phosphatidylinositol-4,5-bisphosphate 3-kinase catalytic subunit alpha
PTEN	Phosphatase and tensin homolog
qRT-PCR	Quantitative real-time polymerase chain reaction
ROS	Reactive oxygen species
SD	Standard deviation
